# Development of a Machine Learning-Based Damage Identification Method Using Multi-Point Simultaneous Acceleration Measurement Results

**DOI:** 10.3390/s20102780

**Published:** 2020-05-14

**Authors:** Pang-jo Chun, Tatsuro Yamane, Shota Izumi, Naoya Kuramoto

**Affiliations:** 1 Department of Civil Engineering, The University of Tokyo, Tokyo 113-8656, Japan; 2 Department of International Studies, The University of Tokyo, Chiba 277-8561, Japan; yamane.tatsuro.20@dois.k.u-tokyo.ac.jp; 3 Department of Civil and Environmental Engineering, Ehime University, Ehime 790-8577, Japan; izumi.shota.17@cee.ehime-u.ac.jp; 4 Yokogawa Techno-Information Service Inc., Tokyo 108-0023, Japan; n.kuramoto@yti.co.jp

**Keywords:** artificial intelligence, machine learning, Random Forest, vibration, damage detection, damage evaluation

## Abstract

It is necessary to assess damage properly for the safe use of a structure and for the development of an appropriate maintenance strategy. Although many efforts have been made to measure the vibration of a structure to determine the degree of damage, the accuracy of evaluation is not high enough, so it is difficult to say that a damage evaluation based on vibrations in a structure has not been put to practical use. In this study, we propose a method to evaluate damage by measuring the acceleration of a structure at multiple points and interpreting the results with a Random Forest, which is a kind of supervised machine learning. The proposed method uses the maximum response acceleration, standard deviation, logarithmic decay rate, and natural frequency to improve the accuracy of damage assessment. We propose a three-step Random Forest method to evaluate various damage types based on the results of these many measurements. Then, the accuracy of the proposed method is verified based on the results of a cross-validation and a vibration test of an actual damaged specimen.

## 1. Introduction

In recent years, the problem of bridge deterioration has become increasingly serious in various parts of the world. For example, in Japan a large number of bridges that were built during the high economic growth period of the 1960s and 1970s are approaching the age of 50 years, which is generally considered the point at which deterioration begins [1,2,3]. In the United States, according to the Infrastructure Report Card by the American Society of Civil Engineers, in 2017 approximately 4/10 of 614,387 bridges were more than 50 years old [4].

**Figure 1:**
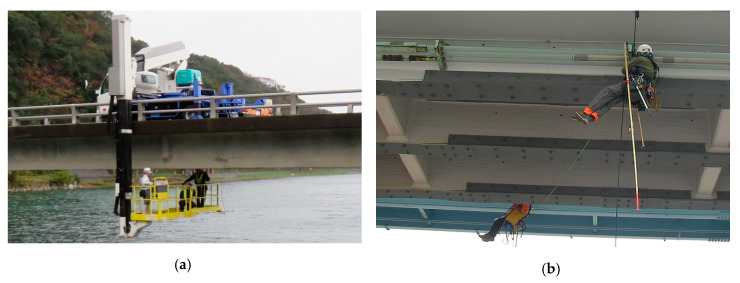
Example of inspection using a high-location work vehicle (**a**) and rope access (**b**).

To prevent the failure of these deficient bridges, it is necessary to conduct appropriate inspections to accurately assess their condition. Typically, in bridge inspections the inspector performs a visual inspection in close proximity to the bridge. Following this, a hammering test is performed in which the bridge is struck with an inspection hammer to determine if there are abnormal sounds. However, when inspecting bridges at high locations, it is not easy to reach them to perform a proximate visual inspection and hammering test. Methods for approaching high locations include, for instance, inspection through rope access or using a high-location work vehicle for the bridge inspection, as shown in Figure 1 [5]. However, considering the large number of bridges, performing these types of inspection would be expensive and would require extensive effort. In addition, there is the issue of large disparities in the results of proximate visual inspections depending on the experience, knowledge, and competence of the inspector. Therefore, such inspection methods lack reliability.

In order to solve these problems, there has been considerable interest in methods that use vibration characteristics, including natural frequencies [6,7,8,9,10], to inspect bridges. Rytter [11] suggested the classification of vibration-based structural damage identification methods as follows:

- Level 1 (detection): determination that damage is present in the structure.
- Level 2 (localization): determination of the geometric location of the damage.
- Level 3 (quantification): quantification of the severity of the damage.
- Level 4 (prediction): prediction of the remaining service life of the structure.

A particularly large number of these studies have focused on changes in natural frequencies, which are summarized in [6,7]. This method is easy because it requires only a small number of measurement points and can be carried out from the bridge itself. However, most of the proposed methods are limited to Level 1, which is the determination of the presence of damage. One of the reasons is that the small number of measurement points cannot provide enough information for the determination of the geometric location of the damage (Level 2) and the quantification of the severity of the damage (Level 3). If we increase the amount of information, we can expect to be able to perform more difficult evaluations that are not limited to judgment of the presence of damage; such examples are given in [12,13]. However, in many cases, even if the amount of information is increased, its interpretation is difficult. This is because the methods, knowledge, and application cases are not yet sufficient to understand the complex effects of damage on vibration. The fact that the method of inspecting structures using vibration has not yet been put into practical use seems to be supporting evidence that there is insufficient understanding of the effect of damage on vibration.

In this study, we perform simultaneous multi-point measurements on aluminum alloy I-beams and analyze not only the natural frequencies but also the maximum value and standard deviation of the response acceleration and logarithmic damping rate at the time of impact. Then, the Level 2 damage localization and Level 3 damage quantification are developed based on these various vibration features.

**Figure 2:**
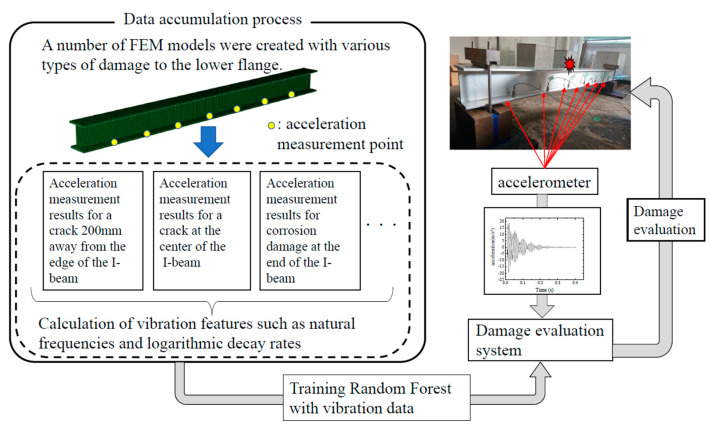
Overview of the methodology of this study.

However, even for such a simple structure, it is currently unclear to what extent the vibration features, such as the maximum response acceleration, are related to the damage. Therefore, in this study we use a supervised machine learning framework, as shown in Figure 2, to identify the location, type, and extent of the damage from the obtained features. Supervised machine learning is a method of learning based on a training dataset consisting of input and output pairs, so that the correct output can be predicted even for input cases whose output is unknown. In this study, we develop an algorithm that outputs the location, type, and extent of damage from the vibration features which are inputs. 

There have been several studies on damage detection and identification using machine learning. For example, in [14] acceleration data on deck and bridge weigh-in-motion data are used as inputs to an artificial neural network to check whether the bridge is being damaged or not. [15] Expressed the damage of the three-story frame structure by gaps between the column and bumper, and increased the number of training data by varying the amount of gaps in small increments. Then, four algorithms—auto-associative neural network, factor analysis, Mahalanobis distance, and singular value decomposition—were applied and their accuracy was compared. Other studies include research using 1D Convolutional Neural Networks [16], support vector machines [17,18,19,20], Gaussian process regression [21], and Genetic algorithms [18,22]. Although these studies propose machine learning methods based on interesting experimental data, they basically remain at the level of damage detection, because it is very difficult to prepare test specimens with various damage geometries in the order of 10^2^ or more in experiments. To solve this problem, [23] uses the finite element method to generate training data and perform numerical simulations including damage patterns. However, in [23] the mode shape measured by the Laser Doppler Vibrometer is the basis for the training data, which is a more difficult measurement condition than the acceleration measurement in this study. In addition, the number of training data is less than 30, which is different from the purpose of this study, which is to improve the accuracy of machine learning based on a large amount of data.

In this study, we propose a method to identify the location of a crack by using Random Forest—which is a kind of ensemble method to achieve a high accuracy prediction using a large number of weak learners—and then evaluating the severity of the damage [24]. Training Random Forest to improve its performance requires a large number of training datasets. However, since it is not practical to obtain data by inflicting various kinds of damage on actual structures, a quantity of data was obtained in this study by the finite element analysis of structures with various locations and degrees of damage. The results of the analysis obtained from a number of finite element models were used to train the Random Forest, and it was confirmed that the damage can be identified with a high accuracy by the leave-one-out cross-validation (LOOCV) method and the actual vibration test of aluminum alloy I-beams.

## 2. Vibration Test of Aluminum Alloy I-Beams

As described above, the damage identification method was developed and validated using aluminum alloy I-beams in this study. In this section, we first provide an overview of aluminum alloy I-beams. Aluminum alloy is used instead of steel because of its good machinability, which makes it easy to simulate damage, while its light weight makes it easy to handle. The following sections describe the vibration test and the finite element model.

### 2.1. Material Properties and Dimensions

**Figure 3:**
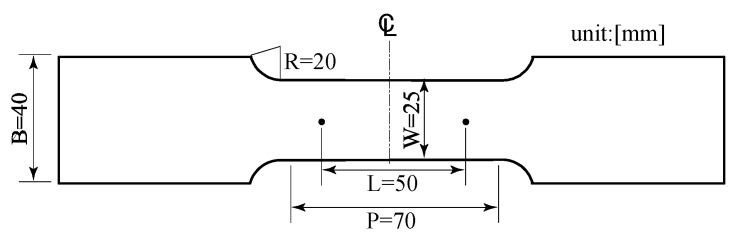
Japanese Industrial Standards No. 5 specimen dimensions.

**Figure 4:**
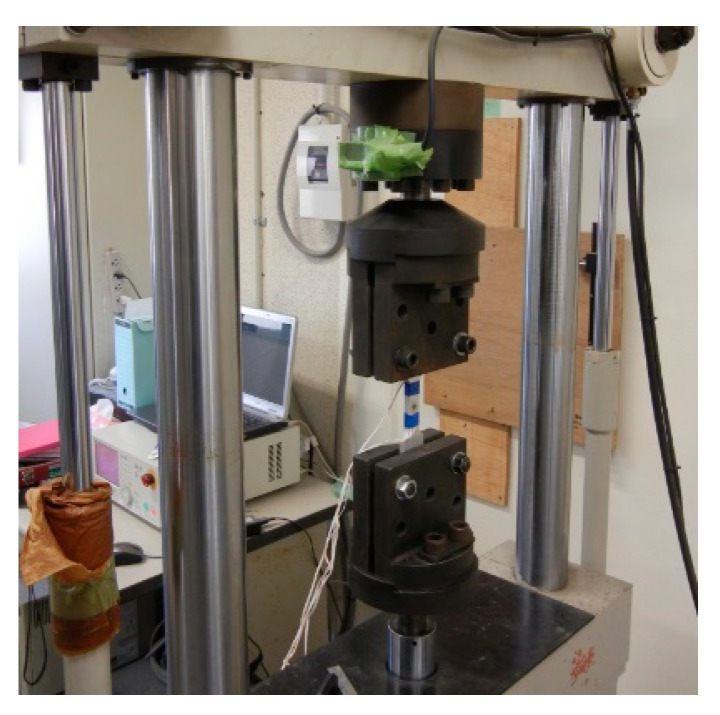
Tensile test of Japanese Industrial Standards No. 5 specimen.

In this study, a general-purpose aluminum alloy (Al-Mg-Si alloy, Japanese Industrial Standards A6061S) manufactured by UACJ Extrusion Corporation was used. The standard mechanical properties can be found in Aluminum Standards and Data [25]. However, in order to obtain more detailed material properties, the Japanese Industrial Standards No. 5 specimen shown in Figure 3 was prepared and subjected to tensile tests by Shimadzu Corporation’s Universal Load Testing Machine with a loading capacity of 10 tonf. The tensile test is shown in Figure 4, and the material constants obtained from the tensile test are shown in Table 1.

**Figure 5:**
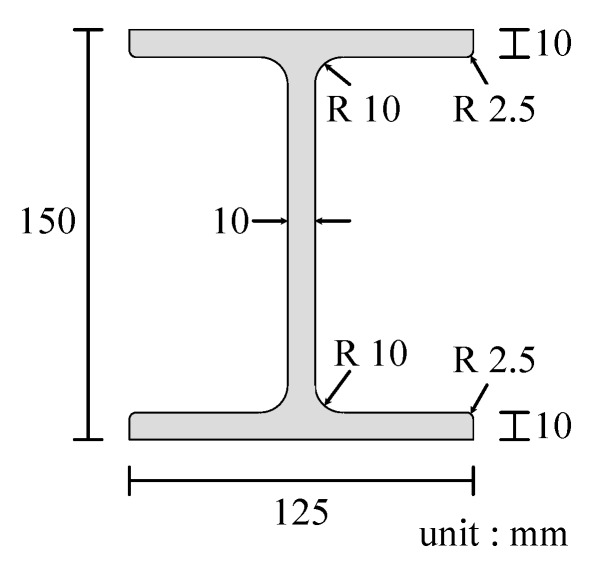
Cross-sectional dimensions of the I-beams.

**Figure 6:**
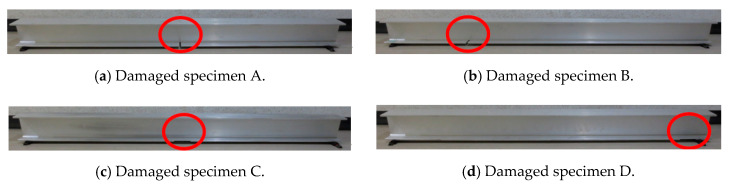
Damaged specimens A to D (The area indicated by the red circle is the damaged area).

The total length and distance between the supports of the beam used in this study were 2000 and 1900 mm, respectively. The cross-sectional dimensions are shown in Figure 5. In this study, we generated a total of five such I-beams, one in sound condition and four damaged. The purpose of the fabrication of these specimens was to investigate the effect of damage on the vibration characteristics and to examine the validity of the damage identification method. In the following description, the undamaged specimen is called the “sound specimen”, and the damaged specimens are referred to as “damaged specimen A” through to “damaged specimen D”. The damage we inflicted on the I-beams was an incision that simulated a crack, and a plate thickness reduction that simulated corrosion thinning on the underside of the lower flange. The size of the incision was 10 mm wide and 125 mm long, while the size of the area subjected to wall thinning was 100 mm wide, 5 mm deep, and 125 mm long. The details of the damage to the damaged specimens A to D are shown in Table 2 and photographic images are shown in Figure 6.

### 2.2. Vibration Experiments

**Figure 7:**
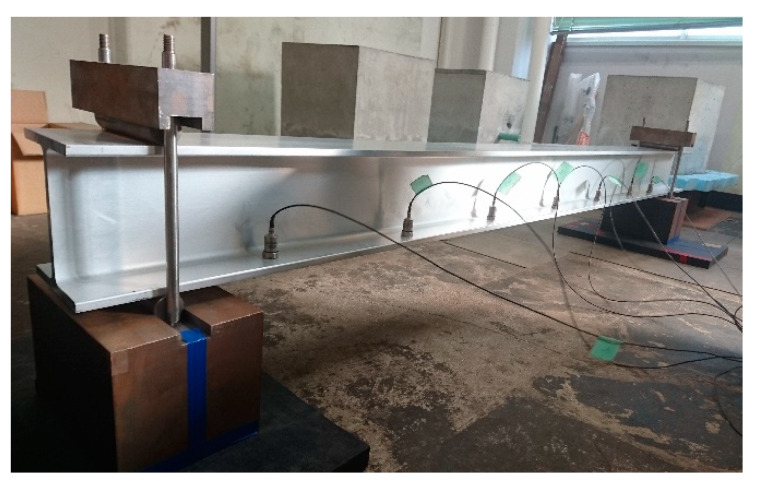
Vibration experiment in this study.

**Figure 8:**
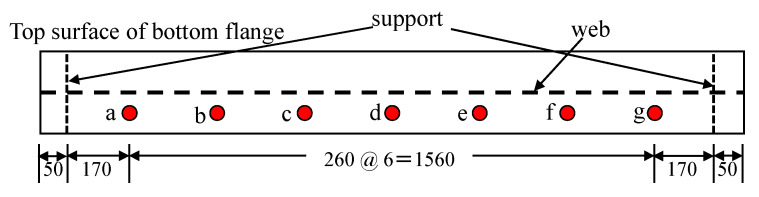
Accelerometer installation position (unit: mm).

**Figure 9:**
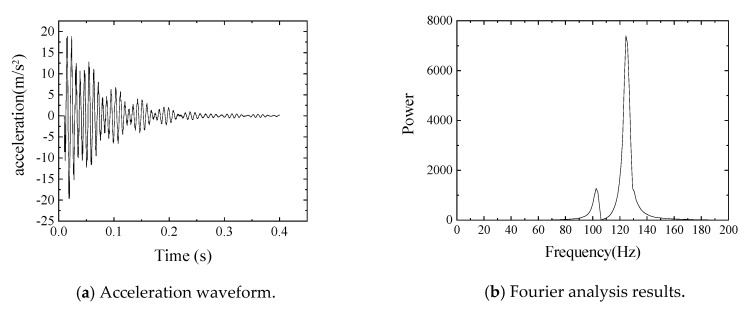
Acceleration waveform and Fourier analysis results of a sound specimen at position d.

In this study, vibration experiments were carried out on both sound and damaged specimens A to D, as shown in Figure 7 and as described in the previous section. A rubber ball (136.10 g) was dropped from a height of 200 mm to the center of the upper flange of this member, causing it to vibrate from the shock. Seven piezoelectric acceleration sensors, MODEL2304A, manufactured by Showasokki Co., Ltd. (Tokyo, Japan) were installed on the lower flange to measure the vibration, as shown in Figure 8. The charge generated by the accelerometer was recorded on a personal computer through an amplifier built-in power supply and an A/D converter (A/D conversion rate of 2 μsec, maximum sampling frequency of 160 kHz, A/D conversion resolution of 16 bits). In this study, the sampling frequency was set to 20 kHz and a finite impulse response filter was applied. As an example of the acquisition of vibration waveforms, the acceleration waveforms and Fourier analysis results of the sound specimen at position d are shown in Figure 9.

In addition to the natural frequency of the structure (124.52 Hz), the vibration mode could be confirmed for all the girders around 102.54 Hz, which may have been due to the influence of the floor and bearings. In addition, a vibration mode around 20 Hz is visible from the acceleration waveform, which was due to the two types of rumbling vibrations mentioned above.

### 2.3. Finite Element Model

**Figure 10:**
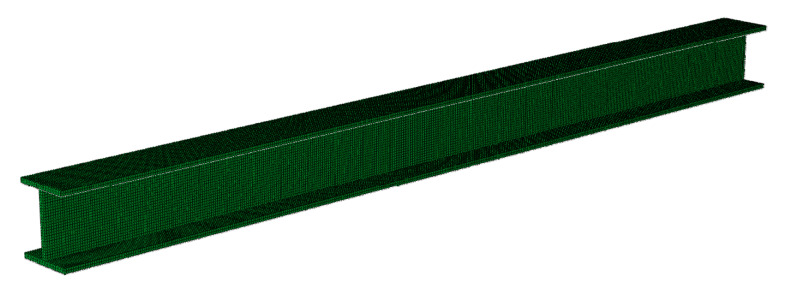
Finite element model of sound specimen.

**Figure 11:**
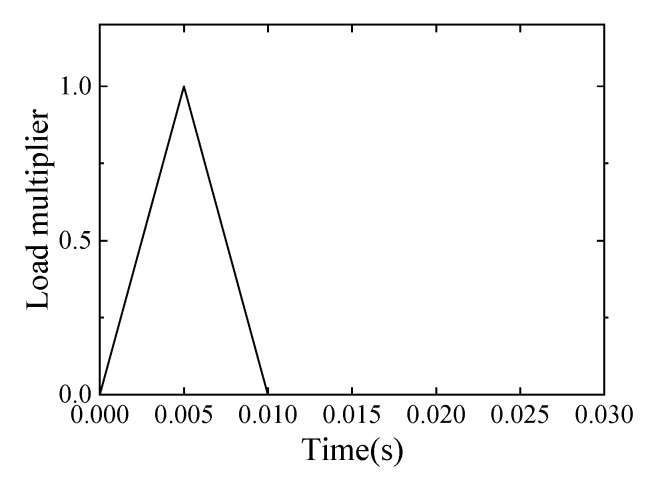
The impact load applied to the finite element model.

In this section, we describe how the vibration experiment in the previous section was simulated as a finite element model. The commercial finite element analysis package Abaqus 6.14 from Dassault Systèmes was adopted for the finite element analysis. Figure 10 shows the finite element model of a sound specimen. The element used in the model was a 3D eight-node isoparametric brick element with a reduced integration (C3D8R). The number of nodes was 97,443 and the number of elements was 62,400. The mesh size was 5 mm × 5 mm × 5 mm. The boundary condition was a simple support at 50 mm from both ends of the lower flange, as in the experiment in Section 2.2. The impact load was applied to the center of the upper flange of this member to replicate the ball drop test shown above. The change in the impact load over time is shown in Figure 11. The time increment in the explicit method was set to 5 μs, the same as the sampling interval in the vibration experiment. This also satisfied the Courant-Friedrichs-Lewy condition.

In order to verify the validity of the model, the primary natural frequency obtained by the finite element model was compared with the primary natural frequency obtained by applying fast Fourier transform (FFT) to the vibration test results of the sound specimens described in the previous section. The finite element analysis result was 124.32 Hz and the actual beam was 124.52 Hz, so the two values were very close to each other, indicating that the finite element model developed here reproduced the experimental conditions adequately.

**Figure 12:**
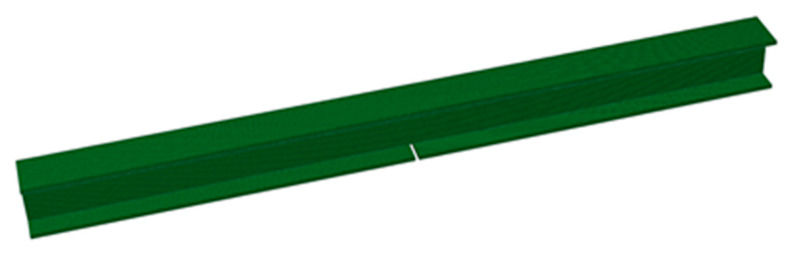
Finite element model of damaged specimen A.

**Figure 13:**
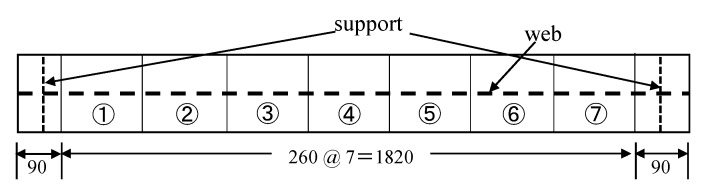
Lower flange area division (in mm).

Another advantage of the finite element model is that it is easy to use it to model damage. An example of a damaged specimen A is shown in Figure 12. In addition to reproducing the actual specimen as shown in Figure 12, it is also easy to create a number of specimens with arbitrary damage and analyze them. These results can be used to train the damage detection system in the lower right part of Figure 2. It would be ideal if these data could be obtained only from the results of vibration tests on actual structures. However, it is difficult to produce a large number of actual damaged members, so we generated and analyzed a number of finite element models. The specific way to inflict damage is as follows. First, the lower flange is divided into seven areas, as shown in Figure 13. In Figure 13, the circled numbers are the area numbers. For the areas that were determined to be subjected to corrosion damage, the area was thinned according to Poisson’s random number in reference to [26,27]. For the areas that suffered crack damage, the location of the crack in the area was randomly determined according to a uniform random number. In this study, 2000 such models were created and a dynamic analysis was performed on each of them.

## 3. Machine Learning Method

As described in the introduction, this study attempts to identify the degree of damage and the presence of cracks in the area shown in Figure 13 using the supervised machine learning framework from the obtained data by the finite element analysis, as explained in the previous section. In this study, we used one of the machine learning methods, Random Forest, which is known for its high accuracy and generalization performance of both classification and regression. In this study, the Random Forest is divided into three steps in order to improve the accuracy. First, the degree of anomaly of the focused area was examined in the first step of the Random Forest, and then the presence of cracks was examined in the second step. Finally, the third step of Random Forest evaluated the degree of damage using the results obtained from the first and second steps. 

In this section, we first give an explanation of Random Forest, followed by a description of the features used as inputs. In structural diagnosis, the presence of cracks obtained from the second step of Random Forest and the degree of damage obtained from the third step of Random Forest are important. The degree of damage was defined as the percentage of cross-sectional loss in the area in the case of corrosion damage and as the ratio of flange width to crack width in the case of cracks in this research.

### 3.1. Random Forest

The authors have already published crack detection [28] and nondestructive inspection methods [29] using decision tree-based algorithms such as Random Forest. In this section, however, the authors will also summarize the theory of Random Forest for the convenience of the reader.

**Figure 14:**
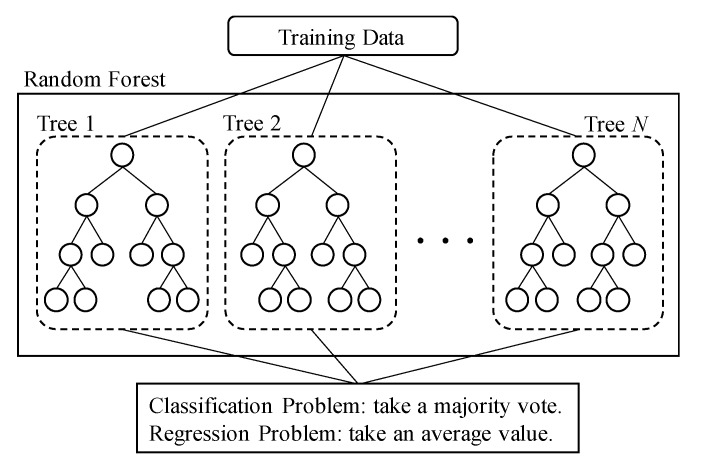
Random Forest.

The Random Forest method is an ensemble method that finalizes results based on the average or majority of a series of decision trees trained from the supervised data. A conceptual outline of Random Forest is shown in Figure 14. In the Random Forest method, each decision tree is constructed based on multiple subsets randomly selected from the original dataset while allowing overlap (i.e., the features randomly selected from bootstrap samples). In this study, the classification and regression trees (CART) method [30] is used for the development of each decision tree. In the CART method, the standard for dividing data at node t is determined so that the decline rate of an indicator called impurity, represented in Equation (1), is maximized. In this case, the Gini coefficient was used as the impurity.

(1) $$\Delta GI(t)=N_{t}GI(t)-N_{L}GI(t_{L})-N_{R}GI(t_{R}),$$

where *GI*(*t*) is the Gini coefficient at node *t*; *N_t_*, *N_L_*, and *N_R_* are the number of items before division, on the left after division, and on the right after division, respectively; and *t_L_* and *t_R_* are the nodes after division on the left and right branches, respectively. The Gini coefficient is defined by Equation (2) as follows:

(2) $$\begin{array}{ll} GI\left(t\right) & =\sum_{i\neq j}p\left(C_{i}|t\right)p\left(C_{j}|t\right)\\ & =\sum_{i=1}^{n}p\left(C_{i}|t\right)\left(1-p\left(C_{i}|t\right)\right)\\ & =1-\sum_{i=1}^{n}p{\left(C_{i}|t\right)}^{2} \end{array}$$

**Figure 15:**
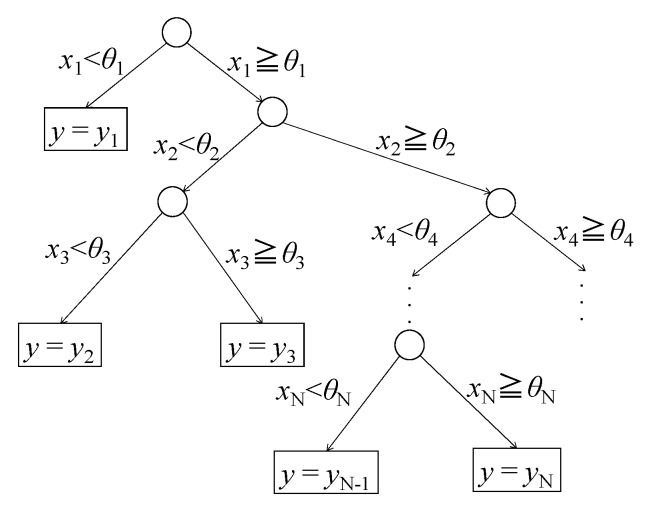
Schematic diagram of the decision tree.

In the above Equation (2), *n* is the number of class and *p* (*C_i_* |*t*) is the probability that the data in class *i* would be selected at node *t*. In the first equation on the right, if we consider the product of probabilities *p* (*C_i_* |*t*) and *p* (*C_j_* |*t*), where class *j ≠ i*, this equation can be interpreted as the error rate for a given node *t*. Thus, with Equation (2), the procedure is designed to branch into decision trees that will be able to reduce this error rate to its lowest value. Figure 15 shows a schematic diagram of the decision tree constructed by the CART method, where *θ_n_* is the reference value of the branch at each node, *x_n_* is the value of the feature at that time, and *y_n_* is the output.

The decision tree developed by the CART method is itself a classifier, but it has a problem of overlearning because the tree is grown by dividing the training data until it is completely allocated. In Random Forest, however, training data extracted by bootstrap sampling and randomly selected features are used to generate individual decision trees, a process which results in a low correlation between individual decision trees. As a result, it is known that the effect of overlearning is very small and that it has the characteristic of increasing the generalization ability. 

It should be noted that the total number of decision trees for all the analyses was increased from five, and it was found that the total number of decision trees converged sufficiently at 300, so the results of all the subsequent analyses show that the total number of decision trees is 300.

### 3.2. Features

The method developed in this study consists of three steps of Random Forest analysis. The first Random Forest determines the degree of anomaly in the target area, the second Random Forest determines the presence of cracks in the target area, and the third Random Forest determines the degree of damage in the target area. The outcome of the complete method is a determination of whether or not cracks exist (second Random Forest result) and the damage rate (third Random Forest result) in the target area. On the other hand, the data given to Random Forest as the input are the vibration features obtained from the waveforms measured by the seven accelerometers. The reason for performing Random Forest three times is to identify complex features, because the effects of cracks and thickness reduction on the vibration are different.

In order to improve the accuracy, it is necessary to select appropriate features as inputs. In this study, by trial and error it was found that the natural frequencies of the first- to third-order bending, the maximum value of response acceleration, the standard deviation of response acceleration, and the logarithmic damping rate were effective for improving the accuracy of Random Forest. They are described below.

#### 3.2.1. The Natural Frequencies of the First- to Third-Order Bending

Natural frequencies are very commonly used parameters for damage detection, as described in the introduction. In the present study, the dominant frequencies from the first to the third-order bending are obtained by FFT from the measurement results. In some previous studies, torsional first-order natural frequencies and/or higher-dimensional natural frequencies were used. However, the relationship between these natural frequencies and the damage is not clear. Therefore, in this study only the natural frequencies of the first- to third-order bending are used. Let this feature be *v*_1_ through to *v*_3_.

#### 3.2.2. The Maximum Value of Response Acceleration

The maximum value of response acceleration is characterized by a larger value near the damage. At the same time, however, it is highly dependent on the magnitude of the impact load, so the ratio to the maximum response acceleration at the center of the flange is calculated to eliminate the effect of the magnitude of the impact load. In this study, acceleration was measured at seven points, as shown in Figure 8, which can be expressed by the following Equation (3).

(3) $$A_{\max}{}_{i}=a_{\max i}/a_{\max4}(i=1,\ldots ,7)$$

In the above equation, *A* denotes the normalized acceleration, *a* denotes the original acceleration, and *i* = 1, …, 7 denotes the measurement result of the acceleration measurement point *a*, …, *g* in Figure 8, respectively. Additionally, the subscript “max” means the maximum value. In this study, acceleration was measured at a total of seven points, but the value at the center of the flange was used for normalization, so that six features are obtained here. Hereinafter, these are referred to as *v*_4_ through to *v*_9_.

#### 3.2.3. The Standard Deviation of Response Acceleration

As the damage increases, the dominant component of the vibration waveform and the response amount itself changes, so there is a relationship between the standard deviation value and the damage. Therefore, the standard deviation of the response acceleration is used in this study. However, since the standard deviation also depends on the magnitude of the impact load as well as the maximum value, the standard deviation of the response acceleration was normalized by the value at the center of the flange, as shown in the following Equation (4), to eliminate its effect.

(4) $$A_{\mathrm{std}\;i}=a_{\mathrm{std}\;i}/a_{\mathrm{std}4}(i=1,\ldots ,7)$$

The subscript “std” in Equation (4) indicates that it is a standard deviation. The standard deviation, as well as the maximum response acceleration, has six features. Hereinafter, these are referred to as *v*_10_ through to *v*_15_.

#### 3.2.4. Logarithmic Decay Rate

From the experiment, it was confirmed that the logarithmic decay rate increased with growing damage and was also changed by the presence or absence of cracks. Therefore, we use the logarithmic decay rate as a feature. Since there are seven acceleration measurement points, let them be *v*_16_ through to *v*_22_.

## 4. Cross-Validation and Accuracy Verification Using Actual I-Beams

In this section, in order to investigate whether the method developed in this study can adequately evaluate the presence of cracks and the degree of damage, cross-validation was carried out and applied to real structures. The specifications of the computer used for this section is as follows: Intel^®^ Core™ i7-10510U Processor@1.80GHz CPU, 16GB RAM, and Windows 10 OS (Santa Clara, CA, USA). 

### 4.1. Cross-Validation

Verification of the validity of the Random Forest model generated in the previous section will be performed by LOOCV in this section. LOOCV is a validation method that will train all the data except for one datum, and a prediction will be made for that one datum [31]. This process was repeated until the rest of the overall data set had been trained. As discussed previously, in this study, 2000 items of data (input-output) were prepared. The Random Forest was trained primarily by using only 1999 items of data because one datum was taken out from the data set to be analyzed. This process was repeated 2000 times until all data sets had been evaluated in order to verify the accuracy of the results.

First, the presence of cracks was investigated by the first and second steps of Random Forest. There were seven areas in each of the 2000 specimens, giving a total of 14,000 areas. Randomly generated cracks were found in 1247 out of 14,000 areas, and no cracks were found in the remaining 12,753 areas. 

The first step of Random Forest is to compute the degree of anomaly from *v*_1_ through to *v*_22_. Then, the result of the first step of Random Forest is set as a new feature, *v*_23_. Next, the presence of cracks is examined by the second step of Random Forest, with 23 features (*v*_1_ through to *v*_22,_ plus *v*_23_) as inputs.

The confusion matrices are shown in Table 3 to show how accurately the presence of cracks was determined by giving *v*_1_ to *v*_23_ as inputs to Random Forest. The percentage of correct answers is very high (98.7%), which indicates the effectiveness of this method.

Next, the degree of damage was derived from the third step of Random Forest and also cross-validated by the LOOCV. In the third step of Random Forest, we added the results of the second step of Random Forest (presence of crack) as *v*_24_ to *v*_1_–*v*_23_, giving a total of 24 features as inputs. In order to evaluate the results, the mean absolute error (MAE), expressed by the following Equation (5), was calculated.

(5) $$\mathrm{MAE}=\frac{1}{n}\sum_{i=1}^{n}\left|{d^{*}}_{i}-d_{i}\right|$$

In the above equation, *n* is the total number of areas 14,000, d^*^_i_ is the true value of the degree of damage, and *d_i_* is the predicted value of the degree of damage. As a result, the MAE is low (1.48%) and can be evaluated accurately. In terms of calculation time, the training time is within the range of 148 s ± 7 s, and the analysis after training is within the range of 0.02 s ± 0.005 s by the computer described above. In order to investigate the effect of the number of data on the accuracy, an analysis was performed for the case where the number of finite element method (FEM) models is 500. In LOOCV, the percentage of correct answer for the crack detection is 98.4% and the MAE for the degree of damage is 2.78%. This result is not much different from the case where the number of FEM models is 2000, and it shows convergence.

**Figure 16:**
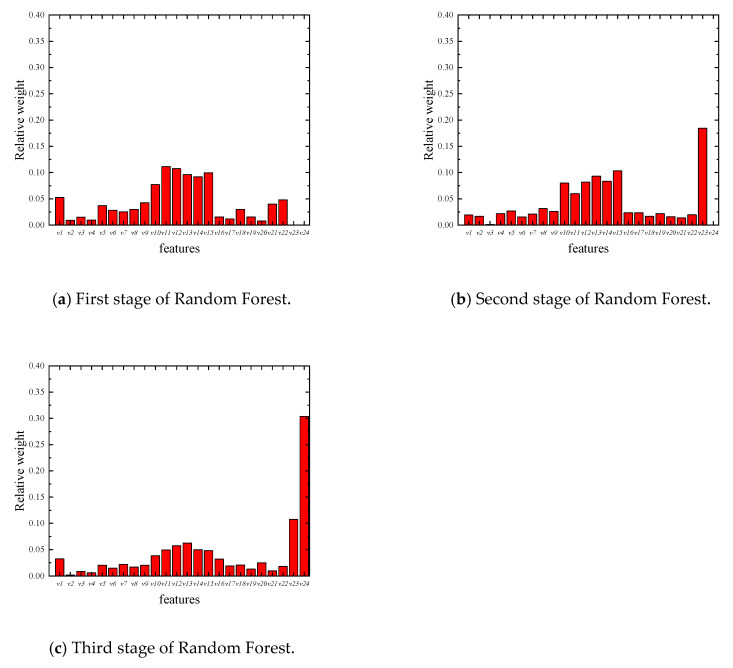
Relative weight of each parameter.

Furthermore, the Random Forest method is capable of weighing the importance of each parameter in its evaluation. The relative weight of each Random Forest parameter can be determined by evaluating the frequency of each corresponding coefficient, which was used as the classification standard in each decision tree, as shown in Figure 16. It can be seen from the figure that the effect of the standard deviation (*v*_10_ to *v*_15_) is significant. The natural frequency of the first order bending (*v*_1_) has a large influence in the first and third stages of Random Forest. This means that although *v*_1_ is less effective in crack detection, it is still important information for thinning detection. In addition, the relative weights of *v*_2*3*_ in the second stage and *v*_23_ and *v*_24_ in the third stage of Random Forest indicate the effectiveness of the three-stage Random Forest analysis.

### 4.2. Accuracy Verification Using Actual Damaged Specimens

In this section, the natural frequencies of the first- to third-order bending, the maximum value of the response acceleration, the standard deviation of the response acceleration, and the logarithmic damping rate are calculated from the vibration test results of the actual damaged specimens A to D shown in Figure 5 and Table 2, and given as an input to the developed damage evaluation system. 

**Figure 17:**
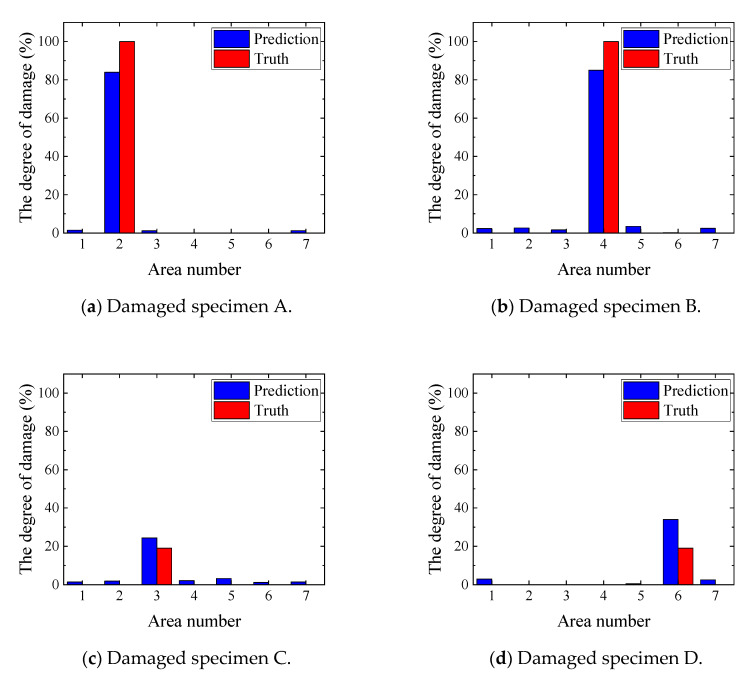
Analysis results of actual damaged specimens.

The results of the analysis are shown in Table 4 and Figure 17. It can be seen from Table 4 that the assessments of whether or not cracks existed are all correct. In addition, it can be seen from Figure 16 that the difference between damaged and non-damaged areas can be accurately assessed.

## 5. Conclusions

In this study, a supervised machine learning system was developed to determine the presence of cracks and the degree of damage by using features calculated from vibration test results. In particular, the novelty of this study lies in the utilization of the Random Forest in three steps. The first step of Random Forest is to find the degree of anomaly of a focused area. Then, in the second step, the results of the first stage of the Random Forest are included in the input features to determine the presence of cracks. Finally, in the third step, the degree of damage is evaluated by including the results of the first and second stages of the Random Forest in the input features to improve the accuracy.

The performance of the developed method is investigated by LOOCV and by considering the results when the method is applied to actual damage specimens; it is shown that the damage could be evaluated with a high accuracy. The results suggest that not only damage detection of beams, but also damage detection of various structures can be achieved by a statistical approach, which will be meaningful in the future when various types of measurement data, in large quantities, will be accumulated.

The future prospects are described below. In this study, aluminum alloy specimens were used for experiments and analyses, but it is necessary to investigate whether a similar method can be applied to steel specimens. However, since the mechanical behavior of the beam in this study was within a linear range, it is basically considered to be possible with steel. In addition, we believe that it would be of great practical value if it could be applied to a complex structure such as a bridge. If we can detect the damage to bridges by a vibration measurement, it will be possible to avoid overlooking the damage during a visual inspection, which could lead to a serious accident [5,32]. Therefore, the authors have started a study on the detection of damage in bridges and have been working diligently on it. In addition, the method of creating many damage patterns, such as this method, is compatible with the stochastic finite element method [33], which treats structures stochastically. By using it, the authors are also working on a study to evaluate the safety of structures stochastically.

## Figures and Tables

**Table 1:** Material properties of the aluminum alloy used in this research.

Elastic Modulus (GPa)	Poisson’s Ratio	Tensile Strength (MPa)	Rupture Elongation (%)	Density (g/cm^3^)
67.0	0.35	197.7	20.1	2.675

**Table 2:** Damage to the test specimens.

Damaged specimen A	Crack 1000 mm from the end.
Damaged specimen B	Crack 500 mm from the end.
Damaged specimen C	Thickness reduction from the end to 950 mm from 1050 mm
Damaged specimen D	Thickness reduction from the end to 1800 mm from 1900 mm

**Table 3:** Crack detection results (cross-validation).

	Ground Truth	No Crack	Crack
Prediction			
No crack		12,632	59
Crack		121	1188

**Table 4:** Crack detection results (actual specimens).

	Ground Truth	No Crack	Crack
Prediction			
No crack		26	0
Crack		0	2
